# Controversies of the assesment and management of polycystic ovary syndrome in adolescents

**DOI:** 10.1186/1687-9856-2015-S1-O19

**Published:** 2015-04-28

**Authors:** Alexia Peña, Preeti Dabadghao

**Affiliations:** 1 Department of Endocrinology/Diabetes, Women’s and Children’s Hospital, The University of Adelaide, Adelaide, SA, Australia; 2 Department of Endocrinology, Sanjay Gandhi Postgraduate Institute of Medical Sciences, Lucknow, Uttar Pradesh, India

## 

The diagnosis of polycystic ovary syndrome (PCOS) in adolescents is difficult as the pathological criteria used in adults like menstrual irregularities, acne, hirsutism and polycystic ovarian morphology could be normal physiological findings during puberty; in addition the syndrome is heterogeneous and there is limited high quality evidence. [1-3] Three international conferences have been held reporting different criteria for diagnosis of PCOS in women [Table 1]. [4-6] The 2011 Australian PCOS evidence-based guideline [1], the 2012 international evidence-based workshop [2] and the 2013 Endocrine Society Clinical Practice Guideline [3] highlight the issues of applying adult criteria to diagnose PCOS in adolescents.

**Table 1:** Diagnostic criteria for PCOS in women

PCOS definition	Clinical (modified Ferriman-Gallway score >8*) or biochemical hyperandrogenemia (elevated total or free testosterone level **)	Oligomenorrhoea (< 6-9 menstrual cycles per year) or oligo-anovulation	Polycystic ovaries on ultrasound (>12 follicles in one ovary or volume >10 cc)
NICHD 1990 [4]	Yes	Yes	

Rotterdam 2003 [5]	Yes	Yes	Yes
	
	2 of 3 criteria		

AE-PCOS 2009 [6]	Yes	Yes	Yes
		
		1 of 2 criteria	

* Ethnicity should be considered when assessing hirsutism

**Testosterone assays, puberty and time of the sample should be considered when reviewing levels.

All criteria require exclusion of other conditions: non-classic congenital adrenal hyperplasia, hypothyroidism, Cushing syndrome, hyperprolactinemia or androgen producing tumours which can cause a PCOS-like picture.

Although diagnosis of PCOS is based on its reproductive manifestations, it is a metabolic disorder. PCOS adolescents are at a high risk of having or developing glucose tolerance abnormalities, dyslipidemia and hypertension. Insulin resistance and the consequent development of hyperinsulinaemia seem to be the central pathophysiological mechanism that links PCOS to its associated metabolic derangements; this can occur independent of weight status. Obesity, which is commonly associated with PCOS, exaggerates insulin abnormalities. Adolescents with PCOS should have evaluation of glucose homeostasis and insulin resistance at diagnosis.

PCOS management should include a multidisciplinary team and should be individualized depending on the predominant complaint and weight status. Lifestyle modifications should be the first line treatment in the presence of overweight, obesity and/or insulin resistance. Metformin can also be added. Cyclical progesterone withdrawn bleed or cyclical oral contraceptive pills are used for menstrual irregularities. Antiandrogens like spironolactone and oral contraceptive pills are used for hirsutism. Permanent treatment with laser or electrolysis is usually advised after a course of antiandrogens.

Various aspects of adolescent PCOS will be discussed based on illustrative cases.
